# Correction: Franz et al. Unraveling a Force-Generating Allosteric Pathway of Actomyosin Communication Associated with ADP and P_i_ Release. *Int. J. Mol. Sci*. 2021, *22*, 104

**DOI:** 10.3390/ijms231911121

**Published:** 2022-09-22

**Authors:** Peter Franz, Wiebke Ewert, Matthias Preller, Georgios Tsiavaliaris

**Affiliations:** 1 Cellular Biophysics, Institute for Biophysical Chemistry, Hannover Medical School, 30625 Hannover, Germany; 2 Structural Bioinformatics and Chemical Biology, Institute for Biophysical Chemistry, Hannover Medical School, 30625 Hannover, Germany; 3 Department of Natural Sciences, University of Applied Sciences Bonn-Rhein-Sieg, 53757 Sankt Augustin, Germany

## Table and Figure Legend

The author wishes to make the following correction to this paper [1]:

In the original article, there were following mistakes:

A common term was incorrectly used in the legend for Table 2. The incorrect parameters “Steady-state ATPase” should be replaced with “Steady-state ATPase Activity”. A parameter was incorrectly assigned in the legend for Table 4. The incorrect parameters “*k′*_+5_*/K′*_5_*K′*_6_” should be replaced with “*k′*_+5_*K′*_5_*K′*_6_”. Parameters were incorrectly assigned in the legend for Figure 5. The incorrect parameters “*K*′_5_*K*′_6_” should be replaced with “1/(*K*′_5_*K*′_6_)”. Parameters were incorrectly assigned in the legend for Figure 6. The incorrect parameters “*K′*_DPA_*k*_+4_” should be replaced with “*k*_+4_*K′*_DPA_”. 

## Error in Table

In the original article, there was a mistake in Table 3 as published. Rate and equilibrium constants, and corresponding values were incorrectly assigned. The corrected Table 3 appears below.

**Table 3:** Transient kinetic parameters and equilibrium constants in the absence of actin.

	Unit	M765^wt^	M765^AA^	M765^GGG^
**ATP binding to myosin ^1^**				
*K*_1_ *k*_+2_	(µM^−1^s^−1^)	0.48 ± 0.01	0.38 ± 0.01	0.38 ± 0.01
*k*_−2_	(s^−1^)	0.79 ± 0.49	0.54 ± 0.24	0.43 ± 0.25
*k*_+3_+*k*_−3_	(s^−1^)	42 ± 1	61 ± 2	106 ± 8
**ADP binding to myosin ^1^**				
*k*_−5_ *K*_6_	(µM^−1^s^−1^)	0.58 ± 0.02	0.38 ± 0.02	0.49 ± 0.01
*k*_+5_	(s^−1^)	1.6 ± 0.1	1.8 ± 0.1	1.9 ± 0.1
*1*/(*K*_5_*K*_6_)	(µM)	2.8 ± 0.2	4.7 ± 0.4	3.9 ± 0.2
**Rate of P_i_ release ^1^**				
*k*_+4_	(s^−1^)	0.02 ± 0.01	n.d.	0.02 ± 0.01

^1^ at 20 °C.

In the original article, there were mistakes in Tables 4 and 5 as published. Rate and equilibrium constants and a unit were incorrectly assigned. The corrected Table 4 and Table 5 appear below. 

**Table 4:** Transient kinetic parameters and equilibrium constants in the presence of actin.

	**Unit**	**M765^wt^**	**M765^AA^**	**M765^GGG^**
**Actomyosin interactions in the absence and presence of ADP**				
***k′*_+A_**	(µM^−1^s^−1^)	0.64 ± 0.03	0.61 ± 0.03	0.61 ± 0.03
***k′*_-A_**	(s^−1^)	0.003	0.004	0.005
***1/K′*_A_^1^**	(µM)	0.0057 ± 0.0005	0.010 ± 0.009	0.011 ± 0.009
***k′*_+DA_**	(µM^−1^s^−1^)	0.12 ± 0.01	n.d.	0.18 ± 0.01
***k′*_-DA_**	(s^−1^)	0.006 ± 0.001	n.d.	0.005 ± 0.001
***1/K′*_DA_^2^**	(µM)	0.063 ± 0.002	n.d.	0.024 ± 0.001
**ATP interactions of actomyosin**				
***K′*_1_ *k′*_+2_**	(µM^−1^s^−1^)	0.24 ± 0.01	0.32 ± 0.01	0.92 ± 0.01
***k′*_+2_**	(s^−1^)	719 ± 22	871 ± 80	923 ± 19
***k′*_−2_**	(s^−1^)	0.08 ± 0.05	0.62 ± 0.05	1.48 ± 0.13
***1/K′*_1_**	(µM)	3200 ± 130	2702 ± 279	883 ± 30
**ADP interactions of actomyosin**				
***k′_−_*_5_ *K′*_6_^3^**	(µM^−1^s^−1^)	>1.0 / >0.74	>1.47 / >2.94	4.7 ± 1 / 3.6 ± 1 ^4^
***1/(K′*_5_*K′*_6_)**	(µM)	135 ± 10 ^5^	34 ± 4 ^5^	3.4 ± 1 ^6^
***k′_+5_***	(s^−1^)	>100 ^7^	>100 ^7^	16 ± 1 ^8^
**P_i_ kinetics of actomyosin**				
***k′*_+4_**	(s^−1^)	3.8 ± 1.1	n.d.	14.4 ± 1.0
***k′*_+4_ *K′*_DPA_**	(µM^−1^s^−1^)	0.04 ± 0.01	n.d.	0.79 ± 0.08
***1/K′*_DPA_**	(µM)	94 ± 32	n.d.	18 ± 3
**Weak-to-strong transition**				
***k′***_weak-strong_	(s^−1^)	1.0 ± 0.01	n.d.	2.6 ± 0.04
**Duty ratio**				
t_strong_/t_total_	%	3 ± 1	<4 ± 1	49 ± 4
Experimental	%	<1	n.d.	35

^1^ Calculated: ***k’*_−A_**/***k’*_+A_**; ^2^ calculated: ***k’*_−DA_**/***k’*_+DA_**; ^3^ calculated from ***k’*_+5_*K’*_5_*K’*_6_**; ^4^ obtained from linear fit of *k*_fast_ of competitive ATP/ADP binding experiment with 25 µM ATP; ^5^ obtained from ADP-inhibition of ATP-induced dissociation; ^6^ obtained from hyperbolic fits of A_slow_ and A_fast_ (Figure 5g); ^7^ estimated form the rate of ADP inhibition of ATP-induced dissociation at excess ADP concentrations; ^8^ y-intercept of hyperbola in Figure 5i; n.d. not determined.

**Table 5:** Parameters used for the estimation of the fractional occupancies of intermediate states.

	**Unit**	**M765** ^**wt**^	**M765** ^**AA**^	**M765** ^**GGG**^
**Equilibrium constants**				
***K′*_DPA_**	(µM^−1^)	0.01	0.01	0.05
***K′*_4_**	(µM^−1^)	0.00001	0.00001	0.00001
***K′*_5_**	-	0.5	0.74	7.14
***K′*_6_**	(µM^−1^)	0.02	0.02	0.02
***K′*_1_**	(µM^−1^)	0.0003	0.004	0.001
***K′*_2_**	-	8988	1405	624
***K′*_TA_**	(µM^−1^)	0.001	0.001	0.001
*K*_3_	-	9.5	9.2	9.6
***K′*_3_**	-	95	92	96
**Rate constants of forward reactions**				
***k′*_+DPA_**	(µM^−1^s^−1^)	10	10	50
***k′*_+4_**	(s^−1^)	3.8	5.0	14.4
***k′*_+5_**	(s^−1^)	235	160	20
***k′*_+6_**	(s^−1^)	1000	1000	1000
***k′*_+1_**	(µM^−1^s^−1^)	100	100	100
***k′*_+2_**	(s^−1^)	719	871	923
***k′*_+TA_**	(s^−1^)	1000	1000	1000
***k*_+3_**	(s^−1^)	38	55	96
***k′*_+3_**	(s^−1^)	38	55	96
**Rate constants of backwards reactions**				
***k′*_-DPA_**	(s^−1^)	1000	1000	1000
***k′*_−4_**	(µM^−1^s^−1^)	0.000038	0.00005	0.000144
***k′*_−5_**	(s^−1^)	470	216	2.8
***k′*_−6_**	(µM^−1^s^−1^)	20	20	20
***k′*_−1_**	(s^−1^)	333300	250000	88500
***k′*_−2_**	(s^−1^)	0.08	0.62	1.48
***k′*_-TA_**	(µM^−1^s^−1^)	1	1	1
*k*_−3_	(s^−1^)	4	6	10
***k′*_−3_**	(s^−1^)	0.4	0.6	1

## Text Correction

There were following errors in the original article. 

A correction has been made to 2. Results and Discussion:

1. Paragraph 8. The incorrect constants “*K*′_5_*K*′_6_” should be replaced with “1/(*K*′_5_*K*′_6_)”.
2. Paragraph 10. The incorrect constants “*K′*_DPA_” should be replaced with “1/*K′*_DPA_”. The incorrect constants “*K′*_DPA_*k′*_+4_” should be replaced with “*k′*_+4_*K′*_DPA_”.
3. Paragraph 11. The incorrect constants “*K′*_DPA_” should be replaced with “1/*K′*_DPA_”.

A correction has been made to 3. Conclusions, Paragraph 1. The incorrect constants “*K′*_5_*K′*_6_” should be replaced with “1/*K′*_5_*K′*_6_”.

The authors state that the scientific conclusions are unaffected. This correction was approved by the Academic Editor. The original publication has also been updated.
